# Relationship of the lobular branch of the great auricular nerve to the tympanoparotid fascia: Spatial anatomy for salvage during face and neck lift

**DOI:** 10.1371/journal.pone.0222324

**Published:** 2019-10-10

**Authors:** Anna Jeon, Heejun Ahn, Chang Min Seo, Je-Hun Lee, Woo Seob Kim, Joo Heon Lee, Seung-Ho Han

**Affiliations:** 1 Department of Anatomy, College of Medicine, Chung-Ang University, Seoul, Republic of Korea; 2 Anatomy Laboratory, College of Sports Science, Korea National Sport University, Seoul, Republic of Korea; 3 Department of Plastic Surgery, College of Medicine, Chung-Ang University, Seoul, Republic of Korea; 4 Area88 Plastic Surgery Clinic, Seoul, Republic of Korea; Medical University of Graz, AUSTRIA

## Abstract

To enable selection of a safer suspension site to use in face and neck lifting procedures, the spatial relationship between the tympanoparotid fascia and the great auricular nerve should be clarified. In this study, we aimed to elucidate the position of the tympanoparotid fascia and the pathway of the lobular branch of the great auricular nerve traversing the tympanoparotid fascia. Twenty hemifaces from non-preserved bequeathed Korean cadavers (5 males, 7 females; mean age, 77.0 years) were dissected to determine the great auricular nerve distribution close to the tympanoparotid fascia of clinical significance for face and neck lift procedures. We observed the tympanoparotid fascia in all specimens (20 hemifaces). The tympanoparotid fascia was located anteriorly between the tragus and intertragic notch. Regarding the spatial relationship between the tympanoparotid fascia and the great auricular nerve, we found the sensory nerve entering the tympanoparotid fascia in all specimens (100%), and the depth from the skin was approximately 4.5 mm; in 65% of the specimens, the lobular branch was found to run close to the tympanoparotid fascia before going into the earlobe. Provided with relatively safer surface mapping to access the tympanoparotid fascia free of the lobular branch of the great auricular nerve, surgeons may better protect the lobular branch by anchoring the SMAS-platysma flap and thread to the deeper superior and anterior portions of the expected tympanoparotid fascia.

## Introduction

The tympanoparotid fascia is a dense, whitish fibrous tissue that arises from the tympanomastoid sulcus and extends to the lateral part of the parotid fascia [1–5]. It has been reported that the tympanoparotid fascia can be an effective and safe anchoring structure for the suspension of the platysma flap in face and neck lifting as well as in certain types of thread lifting [2,3,6]. Remarkable studies have revealed the pathway of the great auricular nerve, which governs the sensation of the periauricular area [7–10]. After the publication of these studies, the prevalence of great auricular nerve damage during face and neck lift surgeries should have been reduced. However, periauricular sensory deficit remains the most common complication after facial rejuvenation.

The lobular branch of the great auricular nerve is now believed to travel adjacent to the tympanoparotid fascia and to selectively govern the sensation of the earlobe [10]. To enable selection of a safer suspension site to use in face and neck lifting procedures, the spatial relationship between the tympanoparotid fascia and the great auricular nerve should be clarified. In this study, we aimed to elucidate the position of the tympanoparotid fascia and the pathway of the lobular branch of the great auricular nerve traversing the tympanoparotid fascia.

## Materials and methods

All cadavers used in the present study were legally donated to Chung-Ang University College of Medicine. The present study was conducted in accordance with the Declaration of Helsinki.

Twenty hemifaces from non-preserved bequeathed Korean cadavers were dissected to determine the great auricular nerve distribution close to the tympanoparotid fascia of clinical significance for face and neck lift procedures. The specimen group consisted of 5 males and 8 females, with an average age of 77.0 years. None of the subjects had any signs of previous facial trauma or surgical scars in the face and neck area.

For the measurements, the intertragic notch, otobasion inferius, and otobasion superius were used as the reference points. A line connecting the intertragic notch point and the otobasion inferius was used as the Y-axis reference line, with the intertragic notch as the starting point for measuring. The horizontal line passing perpendicular to the Y-axis reference line and passing through the intertragic notch point was used as the X-axis reference line. A line connecting the otobasion superius and inferius was the reference line used for measuring the location of the tympanoparotid fascia.

The following measurements were obtained: the x, y-coordinate of the lobular branch of the great auricular nerve entry point into the earlobe; the x, y-coordinate of the lobular branch curve point in the earlobe; the location of the tympanoparotid fascia relative to the reference lines; the depth of the tympanoparotid fascia from the skin; and the depth from the skin of the sensory nerve passing through the tympanoparotid fascia. Furthermore, the entry pattern of the lobular branch was classified according to the Y-axis.

All measurements were obtained using a digital calliper (resolution 0.01 mm, CD-20PSX, Mitutoyo, Japan) in millimetres. Subsequent processing of the data was performed by using Excel spreadsheets (Excel 2016, Microsoft Corp., Redmond, Wash., USA).

### Ethical approval

The methods were carried out in accordance with the declaration of Helsinki and the cadavers were legally donated for the research by Chung-Ang University College of Medicine. None of the transplant donors were from a vulnerable population and all donors or next of kin provided written informed consent that was freely given.

## Results

**Fig 1:**
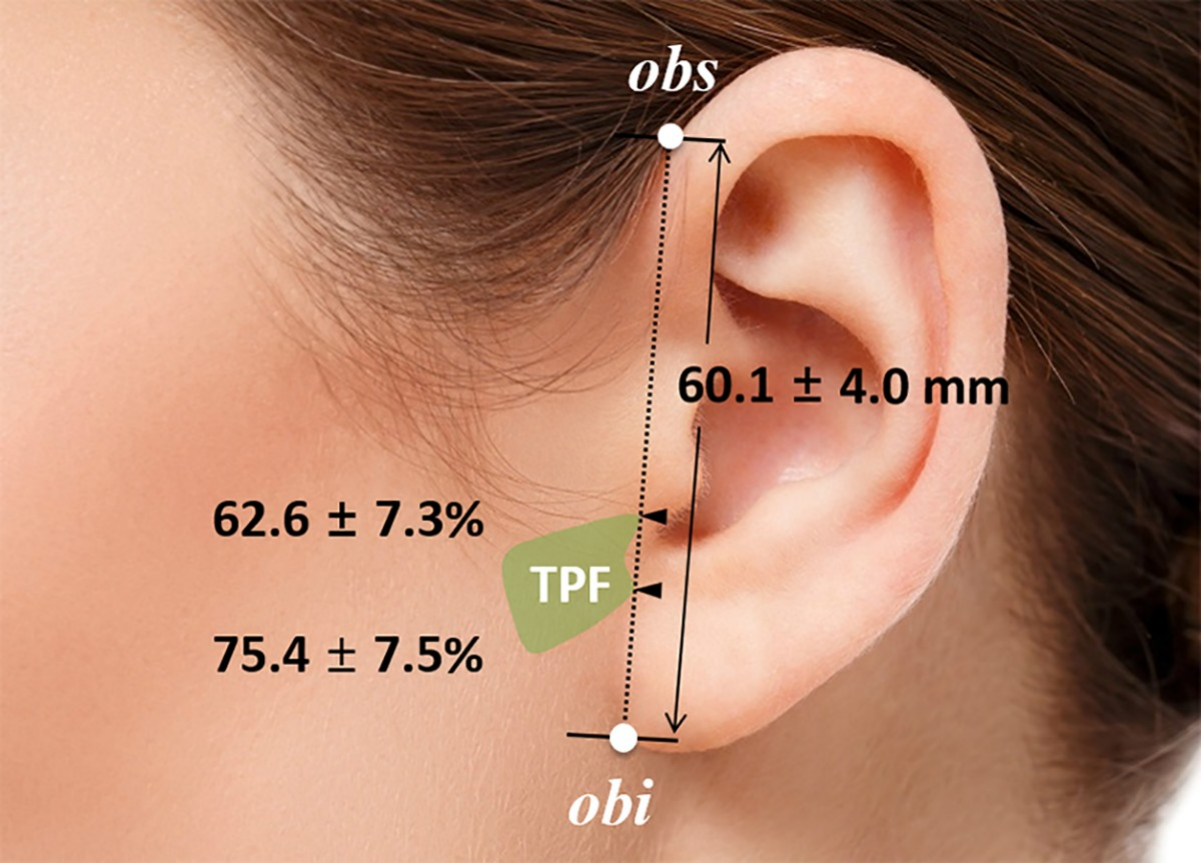
Photograph showing the positional relationship of the tympanoparotid fascia (TPF) with the length between the otobasion superius (obs) and otobasion inferius (obi) points. The mean total length of the reference line from the obs to the obi was 60.1 ± 4.0 mm. The superior and inferior margins of the tympanoparotid fascia were 62.6 ± 7.3% and 75.4 ± 7.5% of the reference line from the obs, respectively. TPF, tympanoparotid fascia; obs, otobasion superius; obi, otobasion inferius.

**Fig 2:**
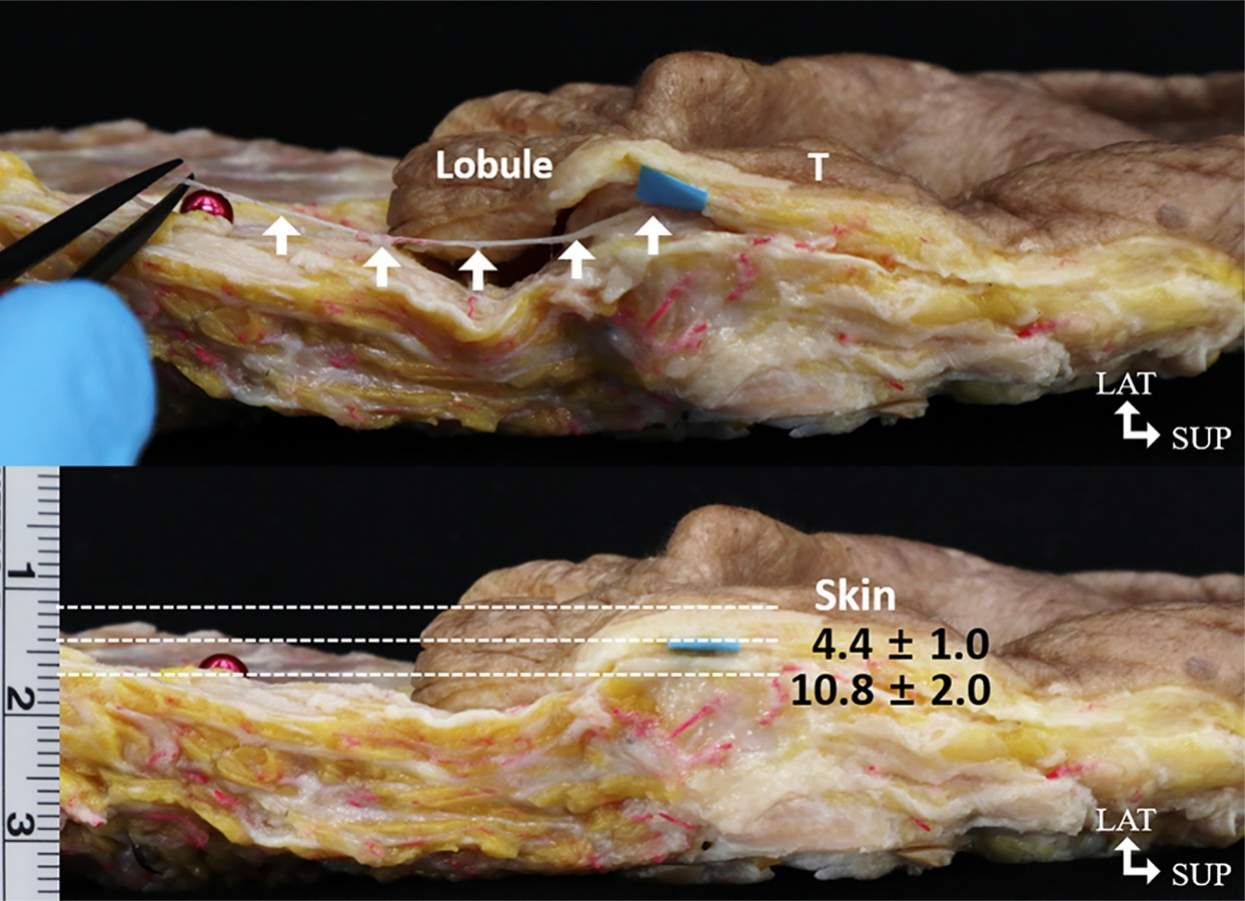
**Photograph of cadaveric tissue specimens showing the relationship of the lobular branch to the tympanoparotid fascia (TPF) and tragus (T) *(Above)*. The lower photograph shows the depths of the lobular branch (4.4 ± 1.0 mm) and TPF (10.8 ± 2.0 mm) from the skin *(Below)*.** *Arrows*: nerves branching out of the GAN into TPF, *Blue square paper*: marking to measure nerves depth at TPF. T, tragus; LAT, lateral; SUP, superior; GAN, great auricular nerve.

**Fig 3:**
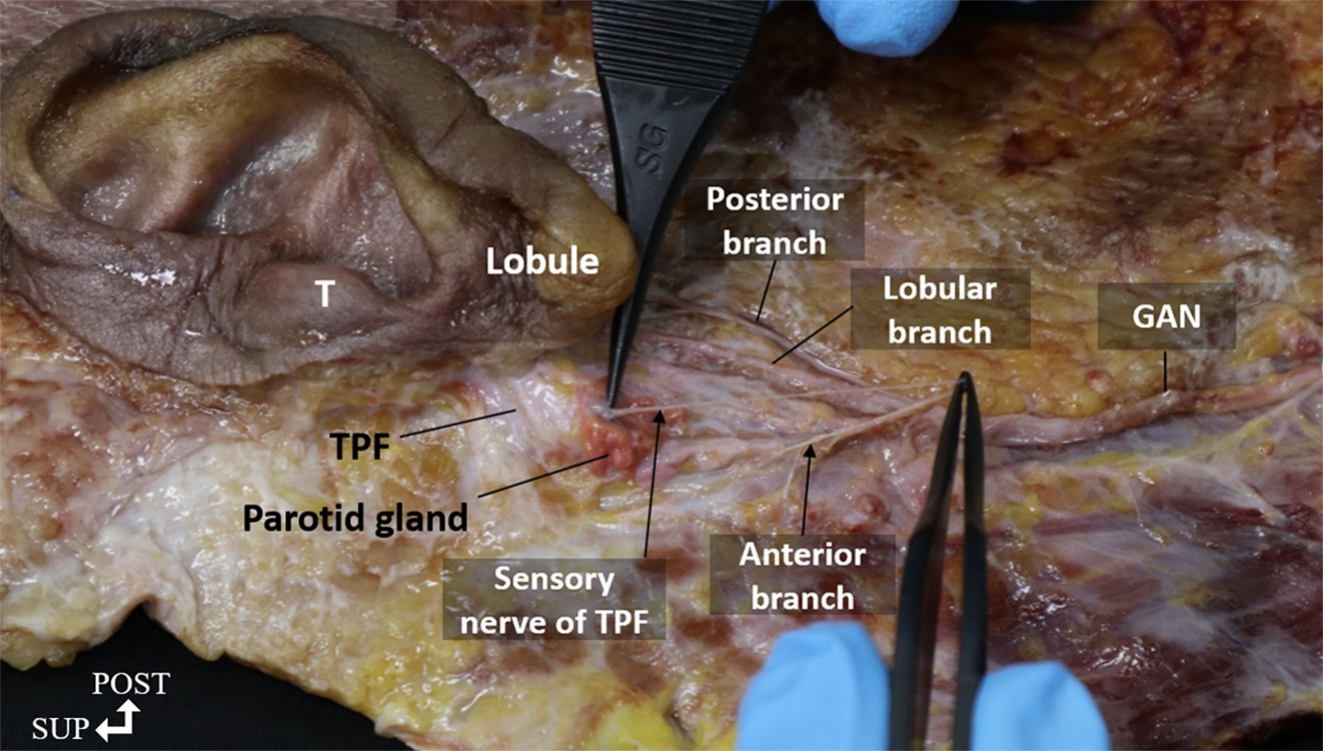
Photograph of a cadaveric tissue specimen showing the great auricular nerve with the tympanoparotid fascia (TPF) from the left side. Anterior, posterior, and lobular branches are shown running to the auricular region. Sensory nerves entering the TPF are bifurcated from the anterior branch. T, tragus; GAN, great auricular nerve; TPF, tympanoparotid fascia; SUP, superior; POST, posterior.

The mean total length of the reference line from the otobasion superius to the otobasion inferius was 60.1 ± 4.0 mm. The superior and inferior margins of the tympanoparotid fascia were 62.6 ± 7.3% (37.4 ± 3.4 mm) and 75.4 ± 7.5% (45.1 ± 3.3 mm) of the reference line from the otobasion superius (Table 1 and Fig 1). The average value of the depth of the tympanoparotid fascia from the skin was 10.8 ± 2.0 mm; the depth from the skin of the anterior branch of the great auricular nerve passing through the tympanoparotid fascia was 4.4 ± 1.0 mm (Table 1 and Figs 2 and 3).

**Table 1:** Mapping of the lobular branch of the great auricular nerve according to the X and Y coordinates.

	X-coordinate	Y-coordinate
Measurements	Mean ± SD (mm)	Mean ± SD (mm)
Total length (Y-axis)		21.6 ± 1.5
Entering point	1.1 ± 2.7	8.9 ± 3.5
Curve point	2.3 ± 1.7	7.7 ± 1.6

Total length, the reference line from the intertragal incisure to the otobasion inferius; SD, standard deviation.

**Fig 4:**
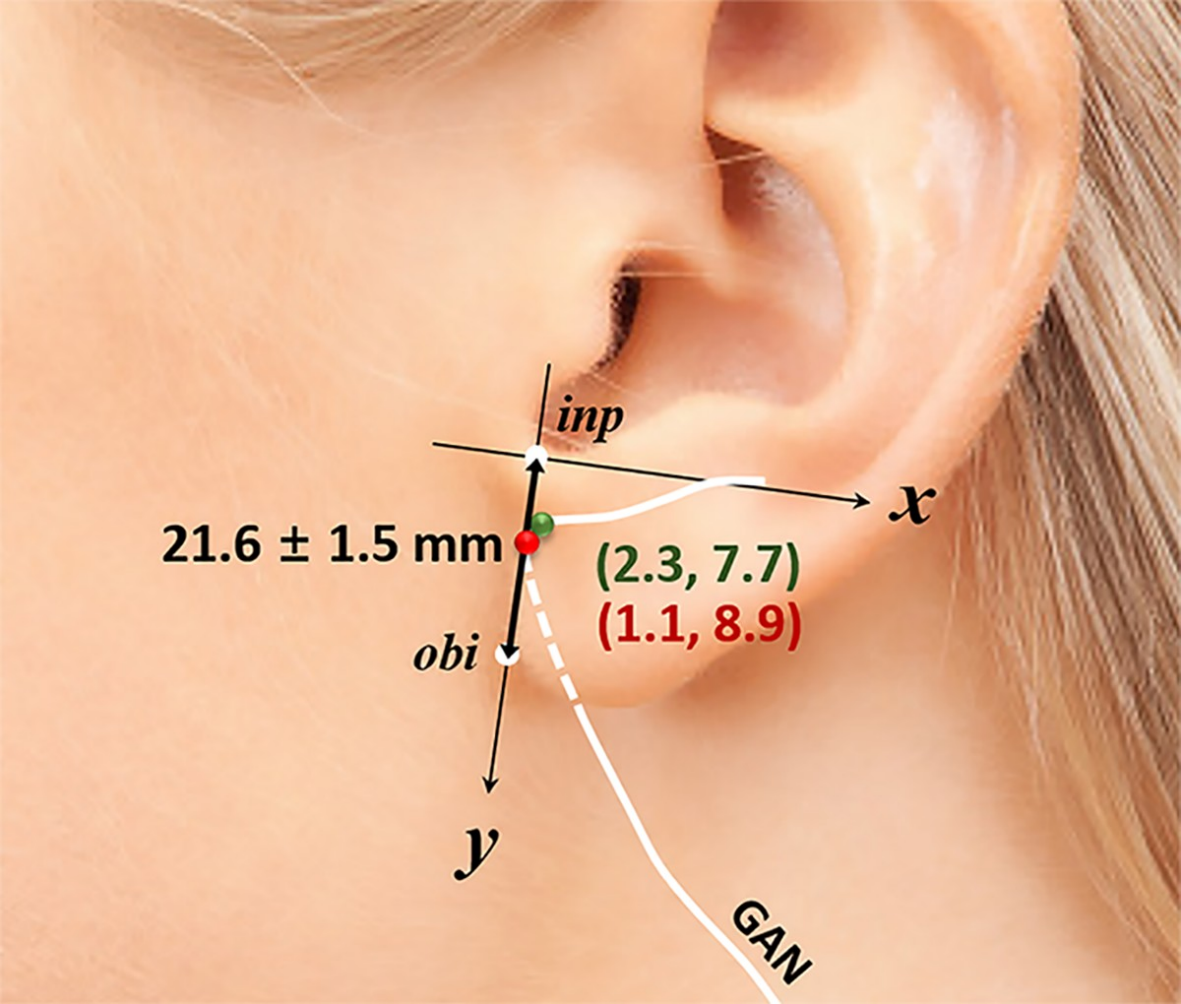
**Entering point (*red*) and curve point (*green*) of the lobular branch on the *x* and *y* coordinates of the earlobe.** The average value of the entering point and the curve point is almost on the reference line (the intertragic notch to the otobasion inferius). inp, intertragic notch point; obi, otobasion inferius; GAN, great auricular nerve.

The mean distance of the reference line, from the intertragic notch point to the otobasion inferius (y-axis), was 21.6 ± 1.5 mm. The average entry point into the earlobe was 1.1 ± 2.7 mm on the x-coordinate and 8.9 ±3.5 mm on the y-coordinate. The average curve point in the earlobe was 2.3 ± 1.7 mm on the x-coordinate and 7.7 ± 1.6 mm on the y-coordinate (Table 2 and Fig 4).

**Table 2:** Mapping of the tympanoparotid fascia.

Measurements	Mean ± SD (mm)
Total length	60.1 ± 4.0
Location of superior border	37.4 ± 3.4
Location of inferior border	45.1 ± 3.3
Depth of TPF	10.8 ± 2.0
Depth of TPF sensory nerve	4.4 ± 1.0

Total length, the reference line from the otobasion superius to the otobasion inferius; Location of superior and inferior borders of the tympanoparotid fascia (TPF), the distance from the otobasion superius; Depth of TPF, the distance between the skin and the TPF; SD, standard deviation.

**Fig 5:**
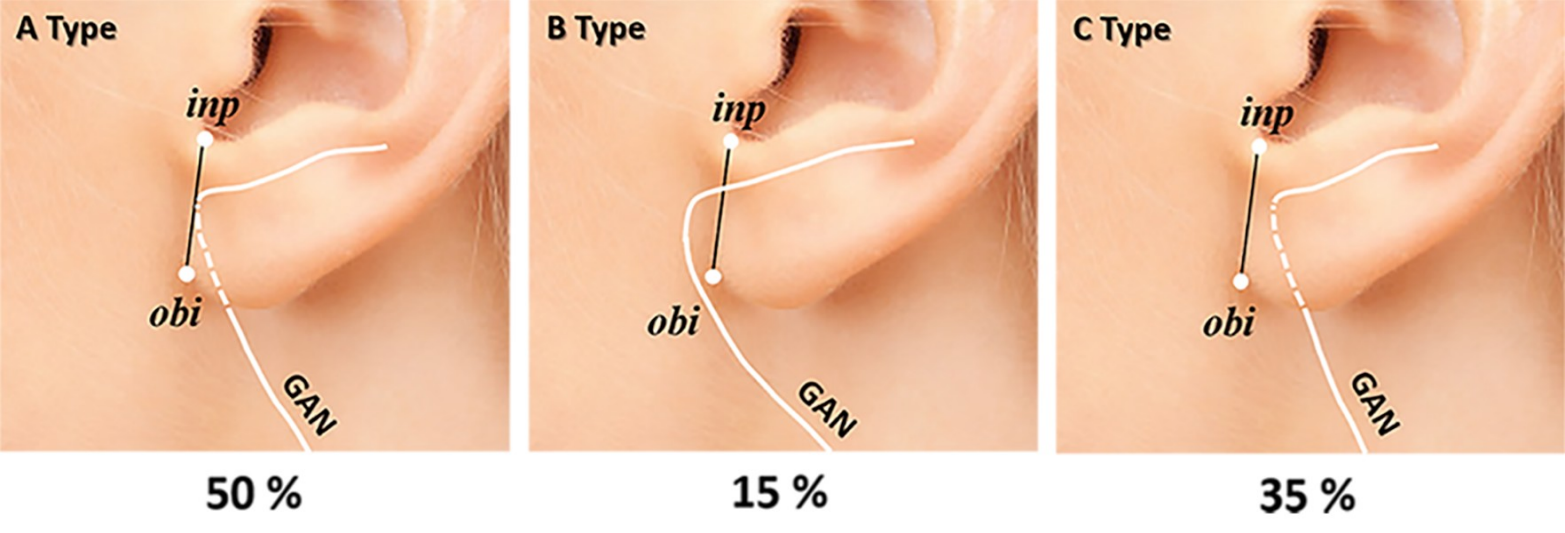
Schematic representation of three entering patterns of the lobular branch into the earlobe. A pattern directly into the reference line (the intertragic notch point to the otobasion inferius) was the most common (50.0%), followed by the laterally entered type (35.0%); the medially entered type was observed least commonly (15.0%). The lobular branch was found to run close to the tympanoparotid fascia before going into the earlobe in 65% of the specimens. inp, intertragic notch point; obi, otobasion inferius; GAN, great auricular nerve.

The entering pattern of the lobular branch was classified into three types according to the spatial relationship with the Y-axis: directly entered, medially entered, and laterally entered. The directly entered type was the most common (50.0%), followed by the laterally entered type (35.0%); the medially entered type (15.0%) was noted in three specimens (Fig 5).

**Fig 6:**
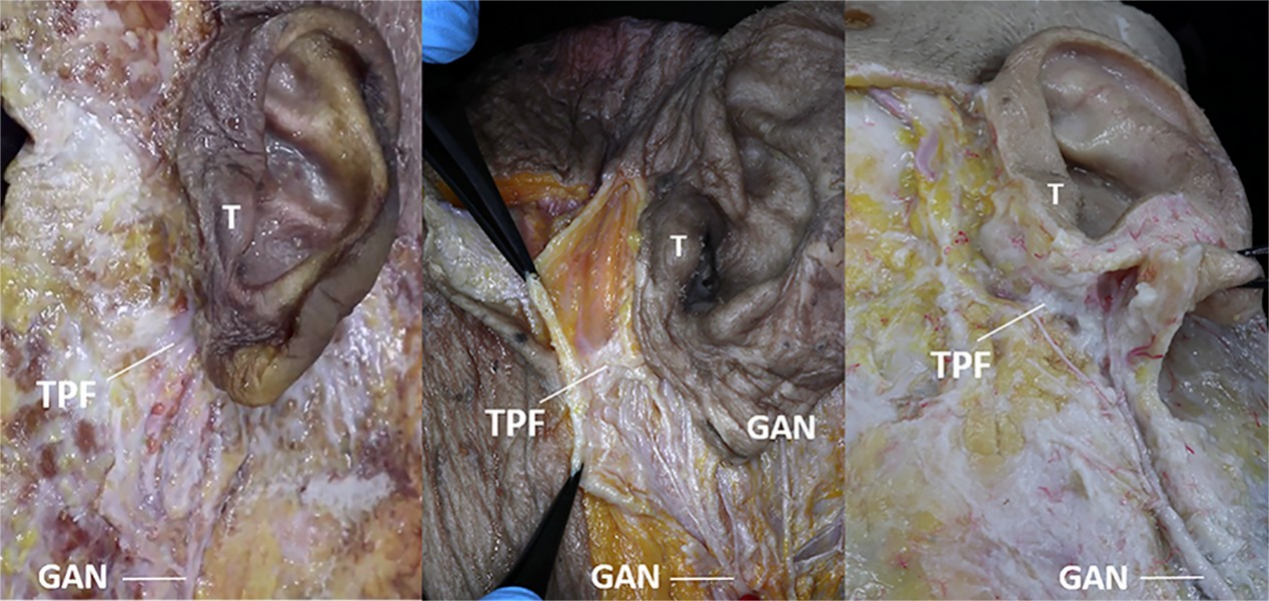
Anatomical study of three non-preserved cadaver hemifaces showing the tympanoparotid fascia (TPF) by dissecting the preauricular region on the left side. **The TPF was found anterior to the intertragic notch and below the tragus.** T, tragus; TPF, tympanoparotid fascia; GAN, great auricular nerve.

We observed the tympanoparotid fascia in all specimens (20 hemifaces). The tympanoparotid fascia was located anteriorly between the tragus and intertragic notch (Fig 6) where the depth from the skin was about 11 mm. Additionally, it was inosculated with the parotid fascia and originated from the auricular cartilage. Regarding the spatial relationship between the tympanoparotid fascia and the great auricular nerve, we found the sensory nerve entering the tympanoparotid fascia in all specimens (20 hemifaces, 100%), and the depth from the skin was approximately 4.5 mm; in 65% of the specimens, the lobular branch was found to run close to the tympanoparotid fascia before going into the earlobe.

## Discussion

The tympanoparotid fascia is known to be a dense, whitish fibrous tissue that arises from the tympanomastoid sulcus to reach the lateral part of the parotid fascia under the superficial muscular aponeurotic system (SMAS) [1–5]. This tympanoparotid fascia was originally proposed by Loré, who termed it a temporoparotid fascia, as a landmark for finding the facial nerve [1]. On revealing its anatomic relationship with the tympanomastoid sulcus, Labbe et al. renamed it the tympanoparotid fascia as well as the honorific Lore’s fascia. They later used this fascia as a fixing point when suturing the lateral end of the platysma-SMAS flap in cervicofacial lifting [2]. With the aid of anatomical studies regarding the position and physical property (tension) of the tympanoparotid fascia, surgeons have commonly used the technique of anchoring the platysma-SMAS flap to this fascia in face and neck rejuvenation. In comparison to anchoring to the mastoid fascia [11], anchoring the cervical platysma-SMAS flap vertically to the tympanoparotid fascia prevents displacement of the earlobe [2], and furthermore, provides a more natural and defined jawline. In facial rejuvenation, vertical repositioning of the platysma muscle is influential, in terms of both the vector of repositioning the SMAS- platysma flap [3,12,13] and maintenance of its anatomic array of platysma muscle fibres [14]. The tympanoparotid fascia is also adopted as an anchoring structure for thread lifting in lower face and neck lift [6].

While outstanding anatomic studies have revealed the anatomy of the great auricular nerve, injury of the great auricular nerve still remains the most common side effect of rhytidectomy, with an incidence of 6% to 7%. Injury of the great auricular nerve results from trauma to the nerve during inexact dissection around the McKinney’s point and posterior auricular area, without a thorough understanding of the passage and spatial anatomy of the great auricular nerve [15–17].

Besides being damaged during dissection, a branch of the great auricular nerve which travels near the tympanoparotid fascia can be damaged from anchoring procedures of both platysma-SMAS flap and certain types of threading to the tympanoparotid fascia. The lobular branch of the great auricular nerve governs the earlobe independently [10]. Earlobe discomfort, pain, paraesthesia, and cold hypersensitivity are previously reported symptoms following injury of the great auricular nerve, specifically due to damage to the lobular branch of the great auricular nerve.

To identify a safer approach to the tympanoparotid fascia that avoids direct injury to the lobular branch of the great auricular nerve, we evaluated the entering pattern of the lobular branch into the earlobe. We found that in 65% of the dissected samples, the lobular branch of the great auricular nerve runs close to the tympanoparotid fascia before entering the earlobe. Excluding the 35% of samples where the lobular branch runs behind the earlobe, the remaining 65% were found to travel adjacent to the imaginary line connecting the intertragic notch point and the otobasion inferius as it exists within the tympanoparotid fascia (Fig 5). The lobular branches travel superficially relative to the depth of the tympanoparotid fascia (Fig 2) and enter the earlobe through the lower half of the tympanoparotid fascia (Fig 4). Provided with relatively safer surface mapping to access the tympanoparotid fascia free of the lobular branch of the great auricular nerve, surgeons may better protect the lobular branch by anchoring the SMAS-platysma flap and thread to the deeper superior and anterior portions of the expected tympanoparotid fascia.
